# Virtual Reality-Based Immersive Rehabilitation for Cognitive- and Behavioral-Impairment-Related Eating Disorders: A VREHAB Framework Scoping Review

**DOI:** 10.3390/ijerph19105821

**Published:** 2022-05-10

**Authors:** Bryan Pak-Hei So, Derek Ka-Hei Lai, Daphne Sze-Ki Cheung, Wing-Kai Lam, James Chung-Wai Cheung, Duo Wai-Chi Wong

**Affiliations:** 1 Department of Biomedical Engineering, Faculty of Engineering, The Hong Kong Polytechnic University, Hong Kong 999077, China; bryan.so@connect.polyu.hk; 2 Department of Computing, Faculty of Engineering, The Hong Kong Polytechnic University, Hong Kong 999077, China; ka-hei-derek.lai@connect.polyu.hk; 3 School of Nursing, The Hong Kong Polytechnic University, Hong Kong 999077, China; daphne.cheung@polyu.edu.hk; 4 Research Institute of Smart Ageing, The Hong Kong Polytechnic University, Hong Kong 999077, China; 5 Centre for Gerontological Nursing, School of Nursing, The Hong Kong Polytechnic University, Hong Kong 999077, China; 6 Sports Information and External Affairs Centre, Hong Kong Sports Institute, Hong Kong 999077, China; gilbert.lam@connect.polyu.hk

**Keywords:** X-Reality, virtual rehabilitation, bulimia nervosa, restrictive food-intake disorder, dysphagia, nutrition

## Abstract

Virtual reality (VR) technology is one of the promising directions for rehabilitation, especially cognitive rehabilitation. Previous studies demonstrated successful rehabilitation in motor, cognitive, and sensorial functions using VR. The objective of this review is to summarize the current designs and evidence on immersive rehabilitation interventions using VR on cognitive- or behavioral-related eating disorders, which was mapped using a VREHAB framework. Two authors independently searched electronic databases, including PubMed, Web of Science, Scopus, CINAHL, EMBASE, and Cochrane Library. Ten (*n* = 10) articles were eligible for review. Treatments for anorexia nervosa and binge eating disorder/bulimia nervosa were reported through enhanced/experimental cognitive behavior therapy (ECT), cue exposure therapy (CET), and body exposure therapy (BET) via the virtual environment. Some studies reported that the VR effects were superior or comparable to traditional treatments, while the effects may last longer using VR technology. In addition, VR was perceived as acceptable and feasible among patients and therapists and could be valuable for supplementing existing therapies, relieving manpower and caregiver burdens. Future studies may consider incorporating haptic, smell, and biofeedback to improve the experience, and thus the effects of the treatments for the users.

## 1. Introduction

Virtual reality (VR) is a category of information technology that creates an interactive and immersive environment for the user through a virtual simulation of the real world [1]. The system often comes with a head-mounted display (HMD) with tracking functions, and a controller using a joystick, or sensor gloves to control the body (avatar) interacting with the virtual environment [2]. It enables the simulated practice of functional tasks at a higher dosage than traditional therapies [3,4,5,6]. It has been reported in various training settings, such as physical training [7], mental/cognitive training [8], and vocation or life-skill training [9,10]. 

Success has been demonstrated in the rehabilitation of motor, cognitive, and sensorial deficiencies using VR [11,12]. VR enables a framework for constant re-enactment, intuitive capacities, and gamification to empower variation and adjustment practices [13]. Besides, training difficulty could be customized based on the abilities and potential of the individuals [14]. The degree of immersion or, in other words, the extent to which the users perceive that they are in a virtual environment rather than the real world is one of the important concepts in VR [15,16]. Immersive VR is generally described as a system that uses a projection onto a head-mounted display (HMD), in which users are represented within the virtual environment, whereas a system with a single-screen projection or desktop display is considered low immersion. Hwang et al. [17] utilized a semi-immersive VR system with a 270-degree touchable screen with locomotor activity and improved cognitive functions and balance among older adults. 

VR-based rehabilitation facilitates a feasible, safe, engaging, and enjoyable medium for patients, especially those with cognitive decline or impairment [18,19]. The strength of VR-based rehabilitation lies in its potential to offer immersive and realistic experiences with multimodal enriched environments that supplement the traditional rehabilitation paradigm’s weakness in realizing training outcomes in daily life [20]. Moreover, some studies reported that the training outcome of VR-based rehabilitation was more long-lasting [20], while better efficacy was found when VR integrated physical and cognitive training components [20]. 

Cognitive and behavioral impairment may result in eating disorders. For example, dementia patients suffer from memory loss, confusion, and ineffective motor coordination. They may fail to eat or self-feed, recognize food, or use tableware [21,22]. Abnormal cognitive behavior, such as cognitive rigidity and obsessiveness, may lead to prototypical compulsive behaviors involving calorie counting, hyperactivity, body checking, and ritualistic eating behavior [23]. Impaired cognitive flexibility with psychosocial risk factors may give rise to anorexia nervosa or bulimia nervosa [24,25]. Different rehabilitation interventions have been proposed, such as education, cognitive-behavior therapy, and mindfulness-based training, despite mixed results [26,27]. Some therapists have taken advantage of VR technology to improve the effectiveness of rehabilitation [28]. 

To this end, the objective of this study is to review the evidence, methods, and performance of VR-based immersive rehabilitation for eating disorders induced by cognitive or behavioral problems. We applied the virtual rehabilitation (VREHAB) model framework [29] to map our review into four themes, including recipients and providers, virtual environment, applications in health care, and social engagement. This framework was previously applied to evaluate virtual reality modalities for health care for the elderly [29] and could be applicable in our current review on virtual rehabilitation for eating disorders, which had similar considerations and constructs. 

## 2. Materials and Methods

A literature search was performed to review articles involving VR-based rehabilitation on individuals with eating disorders resulting from cognitive or behavioral problems. Databases included PubMed, Web of Science, Scopus, CINAHL (via EBSCOHost), EMBASE (via OVID), and the Cochrane Library. The search was conducted using a combination of keywords related to VR technology, cognitive impairment, eating disorders, and rehabilitation. Keywords for VR included “virtual reality”, “virtual healthcare”, “virtual visit”, “immersive reality”, “immersion”, or “haptic”, while those for cognitive impairment and eating disorders were related to dementia, dysphagia, stroke, malnutrition, or other words on feeding and eating disorders. For eating disorders, the search keywords included “eat*” or “chew*” or “swallow*”, while those of rehabilitation included “rehab*” or “train*” or “therapy” (Table S1).

The search was limited to original research articles in English from journals. The inclusion criteria included: (1) cognitive rehabilitation program on eating disorders; (2) immersive VR-based intervention; and (3) human subject data. Studies were excluded if they: (1) applied a non-immersive VR technique; (2) concerned eating disorders unrelated to cognitive or behavioral causes; or (3) had insufficient details on the configuration of VR gear, environment, and playing process. Two authors (B.P.-H.S.) and (D.K.-H.L.) conducted independent searches in November 2021. The first author (B.P.-H.S.) conducted the screening of abstracts and full texts, which was reviewed by the corresponding author (D.W.-C.W.). Any disagreement was resolved by seeking consensus with the other corresponding author (J.C.-W.C.).

**Figure 1:**
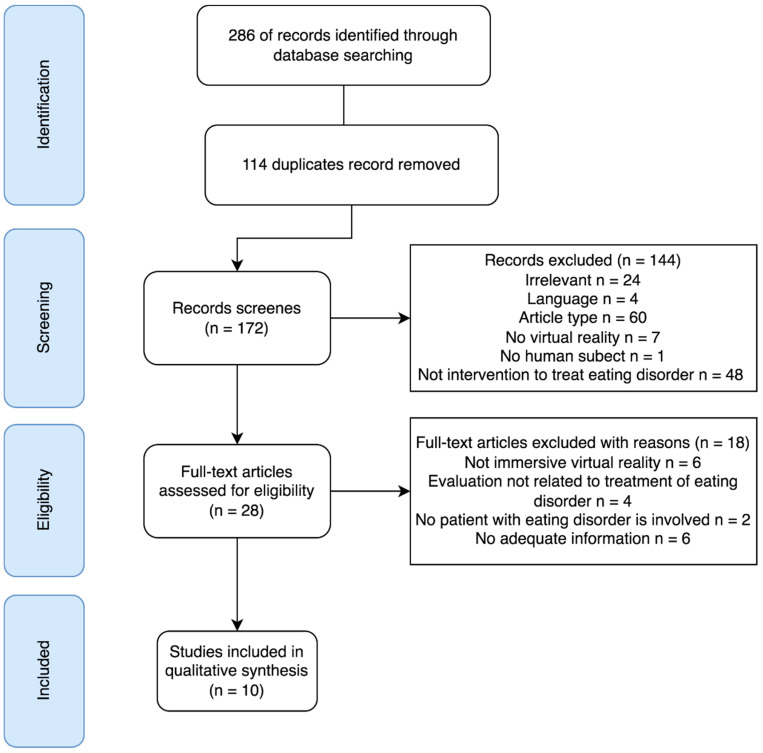
Flowchart of the systematic search and screening process.

The search and screening process for the systematic review is shown in Figure 1. There was no disagreement among authors in the selection of studies for the review. Data synthesis on the eligible articles was conducted based on the VREHAB framework that divided the research themes into recipients and providers, virtual environment, applications in health care, and social engagement, as shown in Table 1 [29]. 

## 3. Search Results

The flowchart of the systematic search and screening process is shown in Figure 1. A total of 172 articles were identified after removing 114 duplicates from the initial search record of 286 articles. After a preliminary screening of the title and abstract, 144 records were excluded because of irrelevancy (*n* = 24), language (*n* = 4), not involving VR (*n* = 7), human subject data (*n* = 1), and rehabilitation programs for eating disorders (*n* = 48). In addition, some articles were excluded because of ineligible article type, such as review, perspective paper, or conference paper (*n* = 60). Twenty-eight articles proceeded to the full-text screening and 18 of them were excluded with the reasons: non-immersive VR (*n* = 6), evaluation not targeted on treatment outcome (*n* = 4), no eating-disorder patients involved (*n* = 2), inadequate information (*n* = 6), such as no evaluation results, and did not specify VR gear and environment designs. Ten studies were eligible for this review.

## 4. Review Theme

### 4.1. Recipients and Providers

Despite our search scope encompassing different cognitive or behavior problems that may be relevant to eating disorders, we only discovered research on BED, BN, and AN. Eight studies involved patients with BED or BN [30,31,32,33,34,35,36,37]. Four studies investigated AN patents [31,33,38,39], while two studies involved a group of patients with unspecified eating disorders [31,37]. We noted that some studies involved both BED/BN and AN groups. Diagnosis or inclusion criteria were confirmed by SCID interviews [40], the DSM-IV-TR, or the DSM-5 scale [41]. 

Binge-eating disorder (BED) is defined as recurrent out-of-control eating without compensatory behavior [42]. It is associated with psychopathological factors. People with BED eat even though not physically hungry and until uncomfortably full. The prevalence of BED was 1.4% [43] and was found to be related to motor disinhibition and impaired executive planning [44]. The investigations on BED and BN were often conducted together, while bulimia nervosa (BN) is a more serious condition, in which patients attempted to compensate for the behavior by self-induced vomiting, purging, or taking laxatives and diuretics [45]. On the other hand, anorexia nervosa (AN) is characterized by abnormal feeding behaviors, primarily because of body image distortion and the pursuit of extreme emaciation [46]. It was estimated that AN affected 4% of the female population [47]. 

A total of 342 individuals were involved, with sample sizes ranging from 1 to 36 (Table 2). Nearly all participants were adults aged 18 to 45, except the Porras-Garcia et al. [34] study, which was a case report on an adolescent aged 15. Only two studies involved both male and female participants [32,38], while the others only recruited female participants. This can be explained by the difference of prevalence between genders in the population (females: AN (0.9%), BED (3.5%); males: AN (0.2%), BED (2%)) [48]. The number of therapist or psychotherapist trainers for VR interventions was similar to that of the traditional interventions.

### 4.2. Virtual Environments and Objects

Immersive VR systems generally include an HMD to display and submerge users into the virtual environment. The HMD device also provides tracking functions for the VR system at different degrees of freedom (DoF). DoF refers to the number of ways that the user can move within a training space. In general, three and six DoF VR systems are available. HMD with 3DoF provides a 360° viewing perspective for users in a panoramic style but fix them at one location, while the 6DoF system allows users to ambulate throughout the environment. 

Three studies created the virtual environment using the software Unity (Unity Technology, San Francisco, CA, USA) [32,34,38], as shown in Table 3. It is a game engine to create 3D or 2D games, as well as interactive simulations and other experiences. Another development platform was the WorldUp software from Sense8 (Tera Software Ltd., Hyderabad, India) [31,33], which could be integrated with Matlab and Simulink now. Besides, Riva’s team utilized the virtual environment from the VR for Body Image Modification 2 (VEBIM 2) (IBM Semea Sud, Naples, Italy) or VR for Eating Disorders Modification (VREDIM) [35,36,37,39]. VEBIM 2 was a virtual environment developed using the VRT 5 engine (Superscape Ltd., London, UK), a software with different libraries for virtual environment development. VEBIM 2 was composed of different environment zones used by the therapists during different training sessions. On the other hand, VREDIM was an enhanced version of VEBIM in which 14 virtual environments were adopted for training. Besides, NeuroVR was open-source software with a predeveloped environment and object library. However, the authors of the paper did not specify which particular environment or objects were chosen from the library for their rehabilitation program [30]. 

The integration of hardware and software in VR could simulate a virtual environment in the first- (1pp) or third-person point of perspective (3pp). 1pp favored the proprioception of embodiment and ownership of the virtual body and enhanced the level of immersion via simulation [49,50]. On the other hand, 3pp facilitated an observer mode that might benefit the rehabilitation of cognitive impairment. Individuals would internalize an objectified self-image when presented with a reference frame in an observer mode and perturb their cognition of body appearance [51]. The treatment principles using the perturbations of body image in patients can be explained by the allocentric lock hypothesis. The hypothesis addressed that patient failed to dislodge their negative body image in their memory and updated from the sensory and proprioceptive inputs in real time [52,53]. VR could unblock the sensory input and facilitate the reinstallation of body image cognition [54]. Table 4 shows the environment and objects used in the virtual rehabilitation.

### 4.3. Application in Healthcare

Generally, there were three types of treatment using the immersive virtual rehabilitation approach, including enhanced/experimental cognitive behavior therapy (ECT), VR-based cue exposure therapy (CET), and body exposure therapy (BET), as shown in Table 4 and Table 5. 

#### 4.3.1. Enhanced/Experimental Cognitive Behavior Therapy (ECT)

ECT was the combination of VR and cognitive behavior therapy (CBT) [55]. ECT aimed at emancipating the negative memory of the body and modifying the associated behaviors and emotions. Before the therapy, existing studies often used the temptation exposure with response prevention protocol for assessment [56] to identify the eliciting factor to abnormal eating behavior. Patients were guided to practice eating, emotional and relational management, and general decision-making and problem-solving skills. In addition, patients were required to rationalize why and what they needed to steer clear of specific emotional and behavioral triggers. This was achieved by different techniques, such as countering, alternative interpretation, label shifting, and deactivating negative beliefs [30,35,36,37,39]. During the treatment, the therapists worked with the patients using the Socratic style using different questions, usually hypothetical and inverse, and from a 3pp to help patients synthesize information and reach conclusions independently. To boost the body experience of the patients, Butter & Cash [57] and Wooley & Wooley [58] integrated therapeutic methods with VR using the body image rescripting protocol, which was a cognitive-, visual-, and motorial-guided imagery approach [59,60].

ECT consisted of four phrases described in the literature [30,35,36,37]. In Phrase 1, an interview was conducted to retrieve the negative perception of body image that shall be attenuated via the design of virtual simulation developed in Phase 2. In Phase 3, the patients re-experienced the triggering events of the negative body image in the virtual environment from 1pp. They were asked to express and discuss their feelings and contemplate the intention to change with gain-framed messages. The main cognitive techniques used here were countering and label shifting. The countering technique initiated whenever distorted perceptions and cognitions are developed. During the discussion about their feelings about the virtual environment, patients attempted to describe their situations with negative emotion word labels that were then listed. After that, the therapist helped the patients replace these emotion labels with two or more other descriptive labels (label shifting). In the last phase, a cognitive reappraisal was conducted with the patients viewing and reassuring themselves in a 3pp through the virtual avatar. In this phase, alternative interpretation and deactivating the illness belief were the two main cognitive techniques used. The patients learned to stop and consider other interpretations of a situation before proceeding to the decision-making stage (alternative interpretation), and the therapist first helped the patients list their beliefs concerning weight and eating (deactivating the illness belief). Another study conducted psychosocial education lessons before the implementation of virtual rehabilitation [31]. The study also guided the estimation of body weight and body image toward the healthy body weight during the virtual rehabilitation section [31]. Marco, et al. [31] highlighted that it is important for ECT to address the attitudinal, perceptive, and emotional construct to achieve a normalization of body image. 

#### 4.3.2. VR Based Cue Exposure Therapy (CET)

CET was integrated with VR technology for virtual rehabilitation. In brief, the patients were exposed to eating triggers in the virtual environment and trained to change their responses. Traditional CET aimed to extinguish the bond between the eating trigger (conditioned stimuli) and the maladaptive binge response (conditioned response) through systematic, gradual exposure [60]. According to the CET model, the craving for food and its metabolic effects could be generated by the conditioned stimuli (e.g., sight, smell, taste, and the context or environment). The presence of the stimuli elicited eating-related anxiety that increased the chance of binge episodes (conditioned response) [61,62,63,64]. Based on the inhibitory learning model, patients could create new associations between the conditioned stimuli and the response to override the existing maladaptive association [65], such as replacing them with safety, self-control, and self-efficacy. The model was optimized when exposure was conducted using various stimuli (in terms of number and type) and across different contexts [60]. Additional out-of-session exposures were arranged as homework in real life to further disassociate the food trigger from the craving response [60].

There are several advantages of VR-based CET over traditional CET. The treatment exposure can be tailored to the fear hierarchy of the patient in the virtual environment to enhance ecological validity. A virtual environment could also encompass a large amount and variety of environmental or object stimuli (e.g., types of food), which can better customize the therapy for each patient and help boost progress [65,66,67] with a perception of safety and control [66]. The novel and exciting VR technology can attract the curiosity of patients to engage and thus improve treatment compliance. 

As prescribed by the Nameth et al. [32] study, for each VR session, the therapist re-assessed the conditions of the eating disorder (e.g., frequency) and reinforced the training components to cope with the eating disorder. The patients were then immersed in the VR environment exposure for a maximum of 30 min. During the exposure, patients were asked to hold and manipulate food, bring the food to the face, and engage other sensory modalities. The level of immersion can also be enhanced by haptic sensation, biofeedback, and food smell, with promising findings previously in physical and cognitive rehabilitation [67,68,69]. The anxiety level over eating control was rated every 45 s in the virtual environment using the visual analogue scale (VAS), and the patients would be brought to the next stage of difficulty when the anxiety rate was reduced to 40%. 

Besides, some studies attempted to incorporate CBT elements into VR-based CET by arranging coping skill practices. For example, changing the physical state of the body (e.g., diaphragmatic breathing and self-soothing via senses), increasing behavioral alternatives to eating, changing the attentional focus, and enhancing motivation to resist cues.

One of the limitations was that the prescription was patient-specific based on the clinical judgement of the therapists. The treatment effects could depend on the experience and expertise of therapists and could be different among patients. Therefore, it was difficult to determine the optimal intensity or level of the VR training. Besides, the implementation of VR rehabilitation involved a start-up cost for the equipment and physician/caregiver training. A cost-effectiveness analysis would be necessary to encourage broader applications, which were previously demonstrated in VR-based pain therapy and post-stroke treatment [70,71]. 

#### 4.3.3. VR-Based Body Exposure Therapy (BET) 

VR-based BET exposed the patients to a mirror in the virtual environment showing the patients their modified virtual body with different BMI. Patients were asked to focus on their different body parts and express their feelings. BET further improved the effects of CBT by targeting the fear of gaining weight (FGW) for AN patients [72,73], and has been successful in reducing FGW and overcoming body dissatisfaction [74,75,76,77]. However, some patients could not comply and maintain their gaze out of fear of the stimulus. VR with eye-tracking function could be implemented to assess whether the patients had placed their gaze on the required body parts. 

According to the Porras-Garcia et al. [34,38] studies, VR-based BET reduced the anxiety level of food and FGW by several steps. In the beginning, the patients were exposed to a virtual avatar of themselves with a higher BMI through a mirror in the virtual environment, which induced the full body illusion (FBI) over the virtual body. FBI, FGW, and anxiety were quantified using the visual analogue scale (VAS). A VR relaxation environment (waterfalls, forests, or beaches) was introduced afterward for debriefing. The VR session ended with a 40% reduction in anxiety compared to the initial level, or after 60 min. In other studies, participants could manipulate body areas on a 3D human figure to represent their body images. In addition, the participants’ actual body images were overlapped with the manipulated 3D human to spot the fitness between human figures. They were also asked to model and discuss their desired body and tested on whether they overestimated her weight after eating [31,33]. 

The virtual avatar of the participants was created and designed using the software Blender [34,38]. Regardless of gender, the avatars wore a white T-shirt, blue jeans, and black trainers. A grey hat covered the hair to reduce any influence of hairstyle on each patient. The avatars also wore an HMD like the patients. Nevertheless, the realism of the avatar was challenged in the aspects of appearance, such as outfit, tone of the skin and hair, etc. Future studies may consider 3D scans and biometric avatars that could improve the realism by personalizing virtual bodies with all the features of the individuals [78]. 

#### 4.3.4. Treatment Effects

Our review demonstrated that VR produced positive outcomes for eating-disorder patients. For binge eating disorders, patients who underwent VR-based ECT reported zero monthly binge-eating episodes when the treatment was completed [30], while the perceived and referenced episodes were also reduced [32]. There was a reduction in body weight after the treatment that continued in the coming year [30]. Besides, VR-based ECT successfully alleviated the contributing psychological factors to binge eating disorders, including body dissatisfaction, anxiety, depressive mood, and low self-esteem [35,36,37]. Patients who completed the treatment demonstrated better engagement in social activities, self-control toward food, and social skills [35,36,37]. 

VR-based BET treatment demonstrated better effects on improving FGW and attentional bias than traditional treatments (control). Better effects also appeared on the fear of putting on weight, estimation of body weight, and body image, though traditional treatments also demonstrated some level of improvement [31,33]. Yet, the attentional bias toward weight-related areas of interest worsened in traditional treatment [38]. In addition, treatment effects in VR intervention were more sustainable, as demonstrated in a 6-month follow-up study on binge eating disorder [36]. Nevertheless, the improvement effects were not superior to traditional treatment [38] and another study also showed that ECT and CBT produced similar improvements in body satisfaction, episodes, and weight recovery [30]. 

As shown in Table 6, there were numerous instruments and parameters for the evaluation of treatment outcome, while most of them were psychological or psychopathological based. Typical instruments included the body attitude test (BAT) [79], situational inventory of body-image dysphoria (SIBID) [80], bulimic investigatory test, Edinburgh (BITE) [81], eating attitude test (EAT) [82], etc. Another review paper included more details on the instruments and techniques to encourage participants to immerse themselves in the process [83]. 

### 4.4. Social Engagement

Virtual rehabilitation using VR was gaining acceptance from both patients and therapists [32,34], despite a medium-to-high nonresponding rate (33.4%) reported by Cesa et al. [30]. Riva et al. [35,37] reported that the VR rehabilitation was safe with minimum side effects such as simulation sickness. Patients commented that VR-based rehabilitation was motivating, helpful, entertaining, and relaxing [38]. Patients also reported that they could feel the stress during “virtual eating” and thus the therapy could mimic a real scenario [33].

However, the high cost of VR gear could be a barrier to implementation [39]. With the improved cost-effectiveness of VR systems in clinical settings [61], we anticipate the popularization of virtual rehabilitation to benefit patients with eating disorders. Table 7 illustrates a list of considerations in social engagement of the reviewed articles. 

## 5. Conclusions

VR technology serves as a supplementary tool in cognitive rehabilitation through realistic simulation of the environment. In addition to the strength of virtualizing the irrealizable scenarios, the virtual environment enables the user to safely experience and participate in different treatments. For example, therapists utilized VR for telemedicine to avoid infection during the pandemic of coronavirus (COVID-19) [84,85]. Besides, VR can design and control past or imaginary experiences in a virtual setting. For ECT and CBT, the therapist asked the patient to imagine different situations using various questions, while VR could facilitate the immersion of the imagination for cognitive therapy. 

In this review, the application of VR in immersive virtual rehabilitation has been demonstrated on AN and BED, which were among the prevalent types of cognitive- or behavioral-related eating disorders and carried more than a five-time increase in mortality [43,47]. CBT was believed to be one of the most promising rehabilitation paradigms [86,87,88] compared to other interventions, such as family therapy [89] and internet-based motivation programs [90]. VR had comparative treatment effects compared to traditional methods, and two studies demonstrated that the effects of VR were sustainable. 

We anticipated some reports on the application of virtual rehabilitation in the treatment of eating disorders associated with dementia, dysphagia, and post-stroke in the review, but in vain. Individuals with dementia often compounded with dysphagia resulting in sarcopenia [91] that mediates the vicious cycle toward anorexia and the risk of aspiration pneumonia [92]. Educational interventions, including changes to the dining environment and table setting [93,94], menu provision, and food service [95,96,97], were often prescribed but were not effective in sustaining the therapeutic effects [98,99,100]. We could not find any attempt to use VR technology to improve the conditions, probably because of the belief that neural death is irreversible and the patient might not be able to train and recover [101,102]. Indeed, previous studies reported that VR intervention could improve cognitive and motor function in older adults with mild cognitive impairment or dementia, especially in attention/execution and memory [103]. Therefore, we believe that VR technology can be one of the alternatives in treating eating disorders among dementia patients, particularly those with signs of forgetting to eat and prolonged mealtimes. 

While the research gap fell in the consideration of dementia and dysphagia, studies on AN, BN, and BED were yet limited. Our search results came up with only 10 eligible articles. Some studies demonstrated the effectiveness in a relatively rigorous study design using a randomized controlled trial [30,31,33,36,37,38]. Furthermore, some articles did not present sufficient details or essential components of the VR gear, systems, or designs. A reporting guideline for VR application in clinical trials in health care was recommended [88], emphasizing the descriptive framework for VR treatment using the TIDIER checklist [104]. We suggested that future studies may incorporate haptics, biofeedback, and olfactory feedback to improve the experience.

There were some limitations in this review. The eligibility criteria could contribute to selection bias in the English journal articles. We noted that social belief, religion, and local culture are associated with diet behavior and may influence eating disorders [105,106,107]. For example, the Mediterranean diet was found to have an inverse association with the development of BED [108]. The language bias in our review may hinder this issue. In addition, obesity was not included in this review since obesity alone is not recognized as an eating disorder [41]. Future studies may consider comparing immersive and non-immersive treatments for eating disorders.

## Figures and Tables

**Table 1:** The themes and components of the VREHAB framework.

Theme	Component
Recipients and Providers	Patients Healthcare Providers Researchers VR Designers
Virtual Environment	3D Tacking System Augmented Reality System Cave System Active Video Game Haptic Interface Simulators
Applications in Health Care	Physical Rehabilitation Cognitive Training Psychosocial Rehabilitation Surgical Simulation Physical Literacy Development
Social Engagement	Accessibility Changing Attitude Cost Cultural Sensitivity Safety Concern

**Table 2:** Basic information of the recipients and providers of the reviewed articles.

Author	Year	Group	Sample Size (M/F)	Age (Mean, SD)	BMI (Mean, SD)	Providers/Trainers
Cesa et al. [30]	2013	VR	0/27	32.9, 8.8	39.2, 5.3	Clinical psy. and psy.ther
		CBT	0/20	29.9, 7.95	41.1, 3.3	
		Ctrl	0/19	32.2, 6.36	41.8, 6.3	
Macro et al. [31]	2013	SEDT	0/9	21.82, 5.75	21.5, 4.28	Eating disorder therapist & co-therapist
		SEDTBI (Ctrl)	0/9			
Nameth et al. [32]	2021	VR	1/10	40.9, 5.7	31.8, 8.1	Therapist recruited from outpatient university clinic
Perpiñá et al. [33]	1999	VR	8 *	18.38, 2.9	21.5, 3.2	-
		SBIT (Ctrl)	5	16.6, 1.3	22.4, 3	
Porras-Garcia et al. [34]	2020	-	0/1	15	18.14	Health psy.
Porras-Garcia et al. [38]	2021	VR	2/14	18.25, 1.30	17.39, 1.06	Psy. & co-therapists
		Ctrl	2/17	19.21, 1.78	17.54, 1.27	
Riva et al. [39]	1998	-	0/1	22	16.8	Clinical psy. & psy.ther.
Riva et al. [35]	2000	BED	0/25	Range: 18 to 45	41.82, 7.81	Clinical psy. & psy.ther.
		Obese	0/18		42.11, 5.43	
Riva et al. [36]	2003	VR CBT Nutritional	0/36	33.07, 8.08	39.80, 6.10	Clinical psy. & psy.ther.
Riva et al. [37]	2004	VR	0/30	33.63, 8.29	39.59, 6.20	Clinical psy. & psy.ther.
		CBT	0/30	32.20, 7.95	41.14, 5.70	
		Nutritional	0/30	33.50, 8.17	39.95, 6.40	
		No action (Ctrl)	0/30	33.16, 8.25	38.59, 6.09	

* Gender not specified; BED: binge eating disorder; BMI: body mass index; CBT: cognitive behavioral therapy; Ctrl: ontrol;c ECT: VR: virtual reality; F: female; M: male; psy.: psychologist; psy.ther.: psychotherapist; SBIT: standard body image treatment; SD: standard deviation; SEDT: standard eating-disorder treatment; SEDTBI: standard eating-disorder treatment with body image intervention.

**Table 3:** Software and hardware for the implementation of VR in the reviewed articles.

Author	Software	Head-Mounted Display	Controller	Tracker/Sensor
Cesa et al. (2013) [30]	Neuro VR open-source software	-	-	-
Macro et al. (2013) [31]	WorldUp, Sense8	V6, Virtual Research	2D mouse	-
Nameth et al. (2021) [32]	Unity, StreamVR	Oculus Rift	Oculus Controllers	Oculus Sensor
Perpiñá et al. (1999) [33]	WorldUp, Sense8	V6, Virtual Research	2D mouse	-
Porras-Garcia et al. (2020) [34]	Unity, Blender	HMD-HTC-VIVE	HMD-HTC-VIVE	HMD FOVE Eye Tracking
Porras-Garcia et al. (2021) [38]	Unity, Blender	HMD-HTC-VIVE	-	FOVE VR-HMD Eye Tracking
Riva et al. (1998) [39]	VEBIM 2	Thunder 400/C VR system		
Riva et al. (2000) [35]	VEBIM 2	Thunder 500/C VR system	Joystick-type input device	-
Riva et al. (2003) [36]	VREDIM	-	-	-
Riva et al. (2004) [37]	VREDIM	Glasstron	Joystick-type input device	Joystick-type input device

VEBIM: VR for body image modification; VR: virtual reality; VREDIM: VR for eating disorders modification.

**Table 4:** Virtual environment and training design of the reviewed articles.

Author	Env. Design	Object/Avatar Design	Interaction/Game Design	Training Context
Cesa et al. (2013) [30]	14 env., including Home, supermarket, pub, restaurant, swimming pool, beach, Gym	2 body images	Practice eating, emotional, relational management, decision-making, problem-solving	Expectation & emotions related to food & weight; Strategies used to cope with difficult interpersonal & potential maintenance situations; Body experience of the subject.
Macro et al. (2013) [31]	5 env. in stage 1 Kitchen in stage 2 Mirror room in stage 3	Virtual scale in stage 2 Mirror and a 3D avatar in stage 3	Distinguish and estimate real, subjective, desired, & healthy weight. Manipulate body areas in the avatar	Psychosocial education on body image distortion, consequences of negative body image, exaggeration in media, cultural factors for body dissatisfaction
Nameth et al. (2021) [32]	4 env., including kitchen, dining room, bedroom, restaurant	30 common binge foods	Access eliciting craving & anxiety about losing control overeating on food & environment	Changing physical state of body; Increase behavioral alternative to eating; Changing the attentional Focus;Enhancing motivation to resist cues.
Perpiñá et al. (1999) [33]	Food area, Exhibition room Mirror room	Virtual scale Posters with different body builds 3D avatar	Estimate body weight after eating a virtual food. Manipulate body areas in the avatar	Education, exposure & cognitive discussion; Learn to correct estimate weight after eating; understand weight as a relative concept
Porras-Garcia et al. (2020) [34]	Simple room w/large mirror on the front wall	-	Asked to observe & focus different part of her virtual body in the mirror	The Virtual body start with the same BMI of the patient and change with progressive increase of BMI until targeted healthy BMI
Porras-Garcia et al. (2021) [38]	Unique room w/o furniture except a large mirror	-	Exposed a silhouette to patients. The patients orally report their throughs & feeling about most of the body parts of their virtual body	Patient exposed to their real silhouette, & processingly increased the BMI of silhouette until a healthy BMI
Riva et al. (1998) [39]	Different rooms of a virtual office	Virtual weighing machine; 7 avatars from underweight to overweight; Panels of model pictures.	Different questions (hypothetical, inverse, & third-person ones) to help patients synthesize information & reach conclusions on their own.	Access & modify patients’ symptoms of anxiety related to food exposure & their body experience
Riva et al. (2000) [35]	Kitchen, closet, bedroom; 4 working env.; Room w/5 doors of different dimensions	Virtual weighing machine; 7 avatars from underweight to overweight; Panels of model pictures; Large mirror showing patients’ real body.	Different questions (hypothetical, inverse, & third-person ones) to help patients synthesize information & reach conclusions on their own.	Access & modify patients’ symptoms of anxiety related to food exposure & their body experience
Riva et al. (2003) [36]	-	-	Different questions (hypothetical, inverse, & third-person ones) to help patients synthesize information & reach conclusions on their own.	Access & modify patients’ symptoms of anxiety related to food exposure & their body experience
Riva et al. (2004) [37]	14 virtual env. including kitchen, bathroom, bedroom, 9 doors room, Shopping mall, Swimming pool & beach)	Body Image VR Scale (BIVRS)	Different questions (hypothetical, inverse, & third-person ones) to help patients synthesize information & reach conclusions on their own.	Access & modify patients’ symptoms of anxiety related to food exposure & their body experience

BMI: body mass index; Env.: environment; VR: virtual reality; w: with; w/o: without.

**Table 5:** Healthcare application in reviewed articles.

Author	Disorder	VR Therapy	Study Design	Protocol
Cesa et al. (2013) [30]	BED	ECT	RCT	VR: 1 × Inpatient program; 5 × group sessions, 1 per week; 10 × 1-h VR sessions, 2 per week. CBT: 1 × Inpatient program; 5 × group sessions, 1 per week; 10 × CBT sessions, 2 per week. Control: Inpatient program
Macro et al. (2013) [31]	AN, BN, EDNOS	ECT + BET	RCT	15 ECT sessions & 8 individual psychotherapy sessions
Nameth et al. (2021) [32]	BED, BN	CET	CS	8 × 1-h session, 1 per week
Perpiñá et al. (1999) [33]	AN, BN	BET	RCT	6 × 1-h session, 1 per week
Porras-Garcia et al. (2020) [34]	BED, BN	BET	CR	5 × 1-h session, 1 per week
Porras-Garcia et al. (2021) [38]	Restrictive AN	BET	RCT	5 × 45-min session, 1 per week
Riva et al. (1998) [39]	AN	ECT	CR	Individual psy. talks for a month, 1 a week; Group sessions, 2 per week; 5 × VR session.
Riva et al. (2000) [35]	BED	ECT	CS	Individual work: Psychometric tests; Weekly supportive psy. talks; 5 × VR sessions. Group work: Psycho-nutritional groups held by nutritionists, 2 per week (for patients eat 1200 kcal per day)
Riva et al. (2003) [36]	BED	ECT	RCT	5 × group sessions aimed at improving assertiveness & motivation to change, 1 per week; 10 × VR sessions, 2 per week.
Riva et al. (2004) [37]	BED, Obese, BN, EDNOS	ECT	RCT	5 × group psy. sessions; 10 × individual VR sessions w/psy. (ECT); 1 × individual diet session. 4 or 6 × group diet sessions;Physical activity

AN: anorexia nervosa; BED: binge eating disorder; BET: body exposure therapy; BN: bulimia nervosa; CR: case report; CS: case series; ECT: enhanced/experimental cognitive behavior therapy; EDNOS: eaters disorder not otherwise specified; Psy.: psychologist; RCT: randomized controlled trial; VR: virtual reality; VR-CET: virtual reality cue exposure therapy; w/: with.

**Table 6:** Evaluation metrics and key findings of the reviewed articles.

Author	Evaluation Timepoint	Endpoint	Instruments	Treatment Findings
Cesa et al. (2013) [30]	Bf-Aft, 1-year FU	Binge Eating Epi.	EDI BSS BIAQ Weight No. of epi.	CBT + VR: - Only effective on weight loss at 1 year. Recurrence: - Occurs in all groups 1-year FU; - Maintains a low rate in VR & CBT.
Macro et al. (2013) [31]	Bf-Aft, 1-year FU	Body Image	BAT, BIATQ, BASS, SIBID, BITE, EAT	SEDTBI: ↑ BAT, BIATQ, BASS, SIBID after treatment and follow-up; and better than SEDT group but not significant.
Nameth et al. (2021) [32]	Bf-Aft	Preliminary Signal of Effectiveness	OBE SBE	↓ Binge epi. (Perceived & Referenced)
Perpiñá et al. (1999) [33]	Bf-Aft	General Psychopathology Measures General Eating Disorder Measures Body Image	BDI, PANAS; EAT, RS, BITE, EDI; BSQ, BIAQ, BAT, BES, BIATQ, ASI, SIBID, BASS, Body Inference, Fear of putting weight	For both groups: ↑ BDI, PANAS, SIBID, BIAQ, BIATQ, BSQ ↓ Fear of putting weight For VR group ↓ Fear of putting weight, ↑ Accuracy in estimating their body weight, closer desired weight to healthy weight; compared to control
Porras-Garcia et al. (2020) [34]	Bf-Aft, 5 months FU	Symptomatology Body Anxiety Body Image Disturbance FBI FGW	BMI EDI PASTAS TSA Body-Related Attentional Bias VAS	After treatment: - ↓ Body anxiety, FGB, VAS; - ↓ Levels of body anxiety & FGW (VAS); - ↑ Patient’s BMI; - ↓ Body dissat., PASTAS, TSA, Complete fixation time & No. of fixations. 5 months FU: - BMI slightly increased; - Body dissat., body image distortion & body anxiety slightly reduced; - ↑ FGW (VAS).
Porras-Garcia et al. (2021) [38]	Bf-Aft, 3 months FU	FBI, FGW Anxiety Level	VAS	↑ FBI, ↓ FGW
		Body Anxiety Attentional Bias	PASTAS	↓ Attentional Bias
Riva et al. (1998) [39]	Bf-Aft	Body dissat.	BSS CDRS FRS	↓ BSS, CDRS, FRS
		Avoidance behav. Grooming habits associated with negative body image	BIAQs	↓ Avoidance behav. & grooming habits associated with negative body image
Riva et al. (2000) [35]	Bf-Aft	Body dissat.	BSS CDRS FRS	BED group: - ↓ Body dissat. (BSS, FRS, CDRS). Obese group: - ↓ Body dissat., better representation of their members.
		Social activities Concealing behav.	BIAQs	Both group: - ↑ Social activities; - ↓ Concealing behav.
Riva et al. (2003) [36]	Bf-Aft, 6 months FU	Anxiety Depressive mood Body Image	STAI BDI EDI 2 BSS CDRS FRS	After treatment: - ↓ Anxiety (STAI) in VR & dietetic groups; - ↓ Depressive mood (BDI) in VR & CBT; - VR amended depressive symptoms; - ↑ BSS, BIAQ, CDRS (VR more effective). 6 months FU: - VR had better efficacy on EDI2, BSS, BIAQ, CDRS.
		Eating control	DIET WELSQ	After treatment: - ↑ DIET, WELSQ except control; - Best results in VR.
		Weight	Weight	After treatment: - ↓ Weight except control. 6 months FU: - ⊗ Weight loss.
		Self-esteem Social skills	RSEQ RAS	↑ RAS in VR; ↑ DIET, except control; Best results in VR.
Riva et al. (2004) [37]	Bf-Aft	Anxiety Depressive mood Body Image	STAI BDI BSS CDRS FRS	Both BED & obese patients: - ↓ STAI in VR & dietetic; - ↓ BDI in VR & CBT; - VR amended depressive symptoms; - ↑ BSS, BIAQ, CDRS more effective in VR.
		Eating control	DIET WELSQ	Both BED & obese patients: - ↑ DIET, WELSQ except control; - Best results in VR.
		Weight	Weight	Both BED & obese patients: - ↓ Weight except control
		Self-esteem Social skills	RSEQ RAS	Both BED & obese patients: - ↑ RSEQ except control; - ↑ RAS in VR.

↑ Significant increase, higher or improve; ↓ significant decrease, lower or deteriorate; ⊗: no significant difference. ASI: appearance schemas inventory; BAT: body attitude test; BDI: Beck depression inventory; Behav.: behavior: BES: body esteem scale; Bf-Aft: before and after; BIAQ: body image avoidance questionnaire; BIATQ: body image automatic thoughts questionnaire; BITE: bulimic investigatory test, Edinburgh; BMI: body mass index; BSS: body satisfaction scale; CDRS: contour drawing rating scale; DIET: dieter’s inventory of eating temptations; dissatis.: dissatisfaction; EAT: eating attitude test; EDI: eating disorders inventory; Epi.: episodes; FBI: full-body illusion; FGW: fear of gaining weight; FRS: figure rating scales; FU: follow-up; OBS: objective binge episode; PANAS: positive and negative affect schedule; PASTAS: physical appearance state and trait anxiety scale; SBE: subjective binge episode; RAS: Rathus assertiveness schedule; RS: restrained scale; RSEQ: Rosenberg self-esteem questionnaire; SIBID: situational inventory of body image dysphoria; STAI: state-trait anxiety inventory; TSA: silhouette test for adolescents; VAS: visual analog scales; W-AOIs: weight-related area of interests; WELSQ: weight efficacy life-style questionnaire.

**Table 7:** Social engagement elements in the reviewed articles.

Author	Construct	Instrument	Finding
Cesa et al. (2013) [30]	Acceptability	Nonrespondent rate	Medium to High (33.4%)
Macro et al. (2013) [31]	-	-	-
Nameth et al. (2021) [32]	Feasibility	No. of therapist attended training after information session & enrolled after the training session; % of participant enrolled; Attendance; % completion; % completed questionnaires by patients & therapists.	High attend & complete rate for both patients & therapists.
	Acceptability	SSQ PQ CSQ-R	SSQ, PQ & CSQ-R indicated general acceptance
Perpiñá et al. (1999) [33]	Acceptability	Patient Feedback	VR system provided an objective judgement that was less resisted by patients. They accepted that they had a body distortion belief
	Attribute	Interview and Patient Feedback	All patients reported 7 to 9 points out of ten for the realism of VR. They felt the stress when ate virtually
Porras-Garcia et al. (2020) [34]	Attribute	Patient Feedback	Motivated, Helpful, Entertaining, Progressively more relaxing Not look realistic enough, clothes look bland, prefer no swimming cap
Porras-Garcia et al. (2021) [38]	Acceptability	Drop-out rates Patient Feedback Author Comment	Low drop-out rates cannot simulate important feature of general appearance Virtual body seemed too young
Riva et al. (1998) [39]	Affordability	Cost	About $12,000 High
Riva et al. (2000) [35]	Safety	Side effects Simulation sickness	Comment that VR had minimum side effects or simulation sickness was found
Riva et al. (2003) [36]	-	-	-
Riva et al. (2004) [37]	Safety	Side effects Simulation sickness	Comment that VR had minimum side effects or simulation sickness was found

SSQ: simulator sickness questionnaire; PQ: presence questionnaire; CSQ-R: client satisfaction questionnaire-revised.

## Data Availability

Data sharing not applicable.

## Supplementary Materials

The following supporting information can be downloaded at https://www.mdpi.com/article/10.3390/ijerph19105821/s1: Table S1: Search Strategy in Literature Review.
