# Supplementary material for: Virtual Reality-Based Immersive Rehabilitation for Cognitive- and Behavioral-Impairment-Related Eating Disorders: A VREHAB Framework Scoping Review
Source: Int J Environ Res Public Health. 2022 May 10;19(10):5821. doi: 10.3390/ijerph19105821 (PMC9141870; doi:10.3390/ijerph19105821)
Supplement: Supplementary file 1 [file ijerph-19-05821-s001.zip › ijerph-1696439-supplementary.pdf]

Supplementary Table S1: Search Strategy in Literature Review

Database	Field	Filter	Search Terms
Web of Science	Topic	Type: Article Language: English	(("deglutition disorders" OR "Deglutition Disorder" OR "Disorders, Deglutition" OR "Swallowing Disorders" OR "Swallowing Disorder" OR "Dysphagia" OR "Oropharyngeal Dysphagia" OR "Dysphagia, Oropharyngeal" OR "Esophageal Dysphagia" OR "Dysphagia, Esophageal" OR "malnutrition" OR "Nutritional Deficiency" OR "Nutritional Deficiencies" OR "Undernutrition" OR "Malnourishment" OR "Malnourishments" OR "Malnutrition, Protein-Energy" OR "Malnutritions, Protein-Energy" OR "Protein Energy Malnutrition" OR "Protein-Calorie Malnutrition" OR "Malnutrition, Protein-Calorie" OR "Protein Calorie Malnutrition" OR "Marasmus" OR "Acute Malnutrition, Severe" OR "Malnutrition, Severe Acute" OR "feeding and eating disorders" OR "Eating AND Feeding Disorders" OR "Feeding Disorders" OR "Disorder, Feeding" OR "Disorders, Feeding" OR "Feeding Disorder" OR "Eating Disorders" OR "Disorder, Eating" OR "Disorders, Eating" OR "Eating Disorder" OR "Appetite Disorders" OR "Appetite Disorder" OR "Anorexia Nervosa" OR "Avoidant Restrictive Food Intake Disorder" OR "Binge-Eating Disorder" OR "Bulimia Nervosa" OR "Diabulimia" OR "Feeding AND Eating Disorders of Childhood" OR "Food Addiction" OR "Night Eating Syndrome" OR "Orthorexia Nervosa" OR "Pica" OR "Relative Energy Deficiency in Sport" OR "Female Athlete Triad Syndrome" OR "Rumination Syndrome") OR (("Dementia" OR "Dementias" OR "Amentia" OR "Amentias" OR "Senile Paranoid Dementia" OR "Dementias, Senile Paranoid" OR "Paranoid Dementia, Senile" OR "Paranoid Dementias, Senile" OR "Senile Paranoid Dementias" OR "Familial Dementia" OR "Dementia, Familial" OR "Dementias, Familial" OR "Familial Dementias" OR "Stroke" OR "Strokes" OR "Cerebrovascular Accident" OR "Cerebrovascular Accidents" OR "CVA (Cerebrovascular Accident)" OR "CVAs (Cerebrovascular Accident)" OR "Cerebrovascular Apoplexy" OR "Apoplexy, Cerebrovascular" OR "Vascular Accident, Brain" OR "Brain Vascular Accident" OR "Brain Vascular Accidents" OR "Vascular Accidents, Brain" OR "Cerebrovascular Stroke" OR "Cerebrovascular Strokes" OR "Stroke, Cerebrovascular" OR "Strokes, Cerebrovascular" OR "Apoplexy" OR "Cerebral Stroke" OR "Cerebral Strokes" OR "Stroke, Cerebral" OR "Strokes, Cerebral" OR "Stroke, Acute" OR "Acute Stroke" OR "Acute Strokes" OR "Strokes, Acute" OR "Cerebrovascular Accident, Acute" OR "Acute Cerebrovascular Accident" OR "Acute Cerebrovascular Accidents" OR "Cerebrovascular Accidents, Acute" OR "cogniti*") AND ("eat*" OR "chew*" OR "swallow*"))) AND ("virtual reality" OR "virtual healthcare" OR "virtual visit" OR "immersive reality" OR "immersion" OR "haptic") AND ("rehab*" OR "train*" OR "therapy")
Scopus	Article Title, Abstract, Keywords	Type: Article Language: English	
EMBASE	Title, Abstract, Author keywords	Type: Article	
CINAHL	/	Language: English	
Cochrane Library	Keyword	/	

Database	Field	Filter	Search Terms
PubMed	/	Language: English	(("deglutition disorders"[Title/Abstract] OR "Deglutition Disorder"[Title/Abstract] OR "Disorders, Deglutition"[Title/Abstract] OR "Swallowing Disorders"[Title/Abstract] OR "Swallowing Disorder"[Title/Abstract] OR "Dysphagia"[Title/Abstract] OR "Oropharyngeal Dysphagia"[Title/Abstract] OR "Dysphagia, Oropharyngeal"[Title/Abstract] OR "Esophageal Dysphagia"[Title/Abstract] OR "Dysphagia, Esophageal"[Title/Abstract] OR "malnutrition"[Title/Abstract] OR "Nutritional Deficiency"[Title/Abstract] OR "Nutritional Deficiencies"[Title/Abstract] OR "Undernutrition"[Title/Abstract] OR "Malnourishment"[Title/Abstract] OR "Malnourishments"[Title/Abstract] OR "Malnutrition, Protein-Energy"[Title/Abstract] OR "Malnutritions, Protein-Energy"[Title/Abstract] OR "Protein Energy Malnutrition"[Title/Abstract] OR "Protein-Calorie Malnutrition"[Title/Abstract] OR "Malnutrition, Protein-Calorie"[Title/Abstract] OR "Protein Calorie Malnutrition"[Title/Abstract] OR "Marasmus"[Title/Abstract] OR "Acute Malnutrition, Severe"[Title/Abstract] OR "Malnutrition, Severe Acute"[Title/Abstract] OR "feeding and eating disorders"[Title/Abstract] OR "Eating[Title/Abstract] AND Feeding Disorders"[Title/Abstract] OR "Feeding Disorders"[Title/Abstract] OR "Disorder, Feeding"[Title/Abstract] OR "Disorders, Feeding"[Title/Abstract] OR "Feeding Disorder"[Title/Abstract] OR "Eating Disorders"[Title/Abstract] OR "Disorder, Eating"[Title/Abstract] OR "Disorders, Eating"[Title/Abstract] OR "Eating Disorder"[Title/Abstract] OR "Appetite Disorders"[Title/Abstract] OR "Appetite Disorder"[Title/Abstract] OR "Anorexia Nervosa"[Title/Abstract] OR "Avoidant Restrictive Food Intake Disorder"[Title/Abstract] OR "Binge-Eating Disorder"[Title/Abstract] OR "Bulimia Nervosa"[Title/Abstract] OR "Diabulimia"[Title/Abstract] OR "Feeding[Title/Abstract] AND Eating Disorders of Childhood"[Title/Abstract] OR "Food Addiction"[Title/Abstract] OR "Night Eating Syndrome"[Title/Abstract] OR "Orthorexia Nervosa"[Title/Abstract] OR "Pica"[Title/Abstract] OR "Relative Energy Deficiency in Sport"[Title/Abstract] OR "Female Athlete Triad Syndrome"[Title/Abstract] OR "Rumination Syndrome"[Title/Abstract]) OR (("Dementia"[Title/Abstract] OR "Dementias"[Title/Abstract] OR "Amentia"[Title/Abstract] OR "Amentias"[Title/Abstract] OR "Senile Paranoid Dementia"[Title/Abstract] OR "Dementias, Senile Paranoid"[Title/Abstract] OR "Paranoid Dementia, Senile"[Title/Abstract] OR "Paranoid Dementias, Senile"[Title/Abstract] OR "Senile Paranoid Dementias"[Title/Abstract] OR "Familial Dementia"[Title/Abstract] OR "Dementia, Familial"[Title/Abstract] OR "Dementias, Familial"[Title/Abstract] OR "Familial Dementias"[Title/Abstract] OR "Stroke"[Title/Abstract] OR "Strokes" OR "Cerebrovascular Accident" OR "Cerebrovascular Accidents" OR "CVA (Cerebrovascular Accident)" OR "CVAs (Cerebrovascular Accident)"[Title/Abstract] OR "Cerebrovascular Apoplexy"[Title/Abstract] OR "Apoplexy, Cerebrovascular"[Title/Abstract] OR "Vascular Accident, Brain" OR "Brain Vascular Accident" OR "Brain Vascular Accidents" OR "Vascular Accidents, Brain" OR "Cerebrovascular Stroke" OR "Cerebrovascular Strokes" OR "Stroke, Cerebrovascular" OR "Strokes, Cerebrovascular" OR "Apoplexy" OR "Cerebral Stroke" OR "Cerebral Strokes" OR "Stroke, Cerebral" OR "Strokes, Cerebral" OR "Stroke, Acute" OR "Acute Stroke" OR "Acute Strokes" OR "Strokes, Acute" OR "Cerebrovascular Accident, Acute" OR "Acute Cerebrovascular Accident" OR "Acute Cerebrovascular Accidents" OR "Cerebrovascular Accidents, Acute"[Title/Abstract] OR "cogniti*"[Title/Abstract]) AND ("eat*"[Title/Abstract] OR "chew*"[Title/Abstract] OR "swallow*"[Title/Abstract]))) AND ("virtual reality"[Title/Abstract] OR "virtual healthcare"[Title/Abstract] OR "virtual visit"[Title/Abstract] OR "immersive reality"[Title/Abstract] OR "immersion"[Title/Abstract] OR "haptic"[Title/Abstract]) AND ("rehab*"[Title/Abstract] OR "train*"[Title/Abstract] OR "therapy"[Title/Abstract])
